# Index Medicus

**Published:** 1879-06

**Authors:** Robert Fletcher

**Affiliations:** Washington, D. C.


### ARTICLE XVII.

WASHINGTON, D. C., May 20, 1879.

*Editors Chicago Medical Journal and Examiner*—GENTN:—In your courteous reference to the *Index Medicus* in the last number of the JOURNAL, you extract from M. Petit's article in *l' Union Médicale* the names which he gives of 25 publications not included in the list of journals, etc., published in the January number of the *Index*. By mentioning merely these supposed omissions I fear you inadvertently do injustice to M. Petit, whose criticism abounds in flattering commendation and generous welcome.

Of the 25 titles not included in our list of 816, I find that 14 were new journals, which had been ordered, but were not received when our January number was compiled. We included nothing in our list which was not actually in our power to index. Ten of these have been since received and indexed, four are yet to arrive. Six other titles belong to publications not within our plan, being 2 dental, 1 educational, and 3 devoted to natural sciences. It was a mistake to mention the *Deutches Archiv für Geschichte der Medicin*, as omitted; it will be found on page 9. Three others, irregular publications, are duly received and indexed, but are hardly to be classed as *current*. Of the remaining two, one is, I suspect, incorrectly described, reports being given for proceedings; the last, alas, was unknown to us. Very truly yours, ROBERT FLETCHER, M.D.

We publish Dr. Fletcher's note, given above, with a great deal of pleasure, and are glad of the opportunity thus afforded of adding a word of explanation. In the copy originally sent by us to the printers, we made a full abstract of M. Petit's remarks, including his really flattering encomium of the *Index Medicus*, which we conceive to be one of the most brilliant and valuable additions ever made to American periodical literature. We stated further, that the suggestions of M. Petit, which were evidently made with the kindest intention, had been without doubt, already considered by the efficient editors of the *Index*.

The whole "matter" of this "copy," in consequence of want of space, was cut down to a mere introduction and the list which followed. It will be remembered by our readers that the portion actually published, was in the form of a "filling," intended merely to complete the page upon which it appeared, hence the accident which may have given to our lines a color they never were intended to produce.

We repeat, that we are glad of the opportunity thus afforded of expressing our estimate of the journal in question. It is certainly one of the most permanently valuable medical publications which appear in any language or in any country. It should be in the hands of every medical gentleman who aims to be a teacher, an author or an expert, and of every one who aims to become thoroughly familiar with the literature of any given medical subject. The *Index* deserves the generous support of the profession of all countries. ED.

---

HEMP SMOKING IN TETANUS. (*Indian Med. Gaz. Aug.* '78.) Mr. Khastagir has successfully treated five cases of traumatic tetanus under the effect of ganja (hemp) smoking. This remedy has long been known, but has fallen into disrepute owing to the use of the hemp by the stomach instead of by smoking, the insufficiency of the doses, and to its being used as an auxiliary to some more potent remedy, and its effects hence neglected. A pipe filled with about 1.0 of dried leaves is kept ever ready near the patient. On every reappearance of a spasm the patient is made to smoke till the leaves are burnt to ashes, on which the muscles of the body instantly relax, the patient shuts his eyes and seems to go to sleep. The pipe is again charged and the advent of the next spasm watched for. In this way the drug has been administered day and night. The longest time which the hemp took to cure was seven weeks, the shortest six days.

Proper attention must of course be given to nutrition and proper evacuation of the bowels. This method has the advantage of being, as it were, self-regulating; the attendant knows when enough has been given as well as when more is needed. It is, however, of course inapplicable for children. (*Practitioner.*)

CORYZA. (*Gazz. Med. Ital., Jan.* '79.) Dr. Rudolphi recommends the use of eucalyptus globulus for the rapid cure of acute coryza or "cold in the head." He has found, by numerous trials on himself and patients, that after chewing a few of the dried leaves and slowly swallowing the saliva, the affection is promptly relieved; often disappearing in the course of half an hour. The remedy is only useful in acute cases. (*Med. Record.*)

